# In Vitro Effects of Sulforaphane on Interferon-Driven Inflammation and Exploratory Evaluation in Two Healthy Volunteers

**DOI:** 10.3390/molecules26123602

**Published:** 2021-06-12

**Authors:** Elena Genova, Maura Apollonio, Giuliana Decorti, Alessandra Tesser, Alberto Tommasini, Gabriele Stocco

**Affiliations:** 1 Institute for Maternal and Child Health IRCCS Burlo Garofolo, 34137 Trieste, Italy; elena.genova@burlo.trieste.it (E.G.); maura.apollonio@burlo.trieste.it (M.A.); decorti@units.it (G.D.); alberto.tommasini@burlo.trieste.it (A.T.); 2 Department of Medical, Surgical and Health Sciences, University of Trieste, 34149 Trieste, Italy; 3 Department of Life Sciences, University of Trieste, 34127 Trieste, Italy; stoccog@units.it

**Keywords:** sulforaphane, type I interferons, *STING*, interferon signature, *GSTM1*

## Abstract

Interferonopathies are rare genetic conditions defined by systemic inflammatory episodes caused by innate immune system activation in the absence of pathogens. Currently, no targeted drugs are authorized for clinical use in these diseases. In this work, we studied the contribution of sulforaphane (SFN), a cruciferous-derived bioactive molecule, in the modulation of interferon-driven inflammation in an immortalized human hepatocytes (IHH) line and in two healthy volunteers, focusing on *STING*, a key-component player in interferon pathway, interferon signature modulation, and *GSTM1* expression and genotype, which contributes to SFN metabolism and excretion. In vitro, SFN exposure reduced *STING* expression as well as interferon signature in the presence of the pro-inflammatory stimulus cGAMP (cGAMP 3 h vs. SFN+cGAMP 3 h *p* value < 0.0001; cGAMP 6 h vs. SFN+cGAMP 6 h *p* < 0.001, one way ANOVA), restoring STING expression to the level of unstimulated cells. In preliminary experiments on healthy volunteers, no appreciable variations in interferon signature were identified after SFN assumption, while only in one of them, presenting the *GSTM1* wild type genotype related to reduced SFN excretion, could a downregulation of *STING* be recorded. This study confirmed that SFN inhibits *STING*-mediated inflammation and interferon-stimulated genes expression in vitro. However, only a trend towards the downregulation of *STING* could be reproduced in vivo. Results obtained have to be confirmed in a larger group of healthy individuals and in patients with type I interferonopathies to define if the assumption of SFN could be useful as supportive therapy.

## 1. Introduction

The mechanisms behind inflammatory diseases are complex and various, making it difficult to find targeted therapies for patients. From the second part of the last century, it was clear that glucocorticoids could represent an effective anti-inflammatory treatment for the vast majority of inflammatory diseases, although burdened by serious adverse effects [1]. However, at the end of the last century, a better knowledge of the pathogenetic mechanisms of these diseases allowed for the development of targeted therapies able to mimic the anti-inflammatory power of cortisone without its adverse effects. For example, following the discovery that some diseases are dominated by the inflammatory effect of specific cytokines, biological drugs usually based on monoclonal antibodies, soluble receptors, or receptor antagonists have been developed [2]. Indeed, anti-tumor necrosis factor-α (TNFα) antibodies have been successfully used in rheumatoid arthritis and in inflammatory bowel disease [3,4], while interleukin-1 (IL-1) inhibitors have found application in autoinflammatory diseases, such as Still’s disease, recurrent pericarditis, gout arthritis, Behçet’s disease, and a set of genetic conditions such as periodic fever [5]. In the last ten years, a set of genetic diseases characterized by a defective regulation of type I interferons production, the so-called interferonopathies, were identified, which often showed poor responses to conventional anti-inflammatory drugs, including biologics and glucocorticoids. Thus, their study allowed to evaluate the potential of novel targeted treatments to reduce interferon driven inflammation [6,7]. Interferonopathies are a heterogeneous group of Mendelian diseases characterized by an abnormal response to nucleic acid stimuli due to either deficiency of nucleases involved in the disposal of nucleic acids, or to defective regulation of downstream effector molecules, leading to excessive production of type I interferon, in particular α and β [6,7]. Interferons are glycoproteic cytokines classified in type I, II, and III according to their cellular origin and receptor structure. Interferons can activate several transduction pathways by different mechanisms resulting in antiviral, immunomodulatory and antiproliferative activities [8,9]. Interferonopathies include Aicardi-Goutières syndrome (AGS), monogenic forms of systemic lupus erythematosus (SLE), *STING*-associated vasculopathy with onset in infancy (SAVI), COPA syndrome and other exceptionally rare disorders [6]. Unfortunately, the measure of type I interferon in human sera is not routinely available in clinical practice, due to the short half-life and the low serum concentrations of the cytokine. Moreover, an isolated interferon dosage may not fully reflect the importance of a prolonged systemic exposure. These issues restrict the ability to diagnose and monitor treatment of these diseases [10]. So far, the assessment of interferon-mediated inflammation in these disorders relies on indirect assays, performed on peripheral blood cells, that present transcriptional changes related to their autocrine or paracrine exposition to high concentration of interferons [11]. One of these approaches consists in the relative quantification of a set of interferon-stimulated genes (ISGs), the so-called interferon signature. One of the most used set of ISGs for interferon signature assessment was proposed by Crow and his group [10], who defined the over-expression of six ISGs (*IFI27*, *IFI44L*, *IFIT1*, *ISG15*, *RSAD2*, and *SIGLEC1*) in a cohort of AGS patients compared to healthy controls. The interferon signature intensity is provided by the calculation of an “interferon score” (IFN score) as the median fold change of the six target ISGs. The validation of this score for the detection of monogenic interferonopathies made it preferred by centers involved in the screening and diagnosis of these rare conditions.

Currently, no targeted drugs for type I interferonopathies are authorized for routine clinical use and the few treatments available control principally the downstream effects of interferons [6,7]. Only a few drugs, such as antimalarial agents, Janus Kinase inhibitors, mycophenolate mofetil, and high dose glucocorticoids, have proven to demonstrate some efficacy. Moreover, anti-interferon α antibodies resulted not useful in the clinical practice while, more recently, antibodies blocking the common type I interferon receptor have been developed and are used with promising results in SLE [12]. To identify more efficient targeted drugs, and for developing drug classes already proven as partially efficient, it is important to develop or improve in vitro models already available reproducing significant disease-related pathogenic mechanisms [13,14]. During the research of new effective treatments, it is also crucial to face the possibility of the infectious risk connected to excessive suppression of cytokines and organism signaling pathways [15]. This implies that active compounds interacting with these inflammatory mechanisms should be modular in action intensity and should be selective, in order to reduce adverse effects.

Historically, medicinal chemistry finds a landmark in plants as a starting point for drug development. In the last years, the field of nutraceuticals has expanded, providing treatments that maintain the bioactive plant compound as close to its native state as possible [16,17]. In this context, sulforaphane (SFN), a bioactive molecule contained in cruciferous vegetables (e.g., broccoli), emerges as a potential phytochemical compound, able to produce positive results in conditions lacking satisfactory pharmaceutical compounds [18], by modulating an important key-component of interferon pathway production. Despite being a food-derived molecule, SFN can reach an intracellular concentration sufficient to affect gene expression, thanks to its high bioavailability [17]. In particular, based on the in vitro effect on cells from patients with SAVI syndrome, SFN may be a reasonable supplement in the treatment of patients affected by type I interferonopathies. Indeed, it is well-known that SFN is an effective activator of the transcription factor NRF2 (nuclear factor erythroid 2-related factor 2), which can modulate key components of the cellular defense processes, operating on redox- and inflammation-regulating gene expression via activation of the antioxidant responsive elements axis [19]. Cytosolic NRF2 contrasts the activity of nuclear factor NF-κB, which drives immune responses to cellular challenges such as bacterial and viral infection and inflammation [19]. As regards the interferon cascade, NRF2 activation driven by SFN leads to downregulation of *STING* (stimulator of interferon genes), an important kinase implicated in type I interferon production, through a mechanism that brings to its mRNA instability in a time and dose dependent manner [20]. In addition, SFN has been demonstrated as an important agent in the regulation of functionalizing (phase I) and conjugating (phase II) xenobiotic biotransformation enzymes [21]. Among phase II enzymes, GSTs are known to be induced by SFN through the activation of the antioxidant responsive elements axis thanks its sulphur interaction with thiol groups of the Keap1 cysteine residues [22]. In addition, GSTs, and in particular the *GSTM1* isoform, play an important role in enzymatic formation and cleavage of the GSH conjugates of isothiocyanates, contributing to SFN pharmacokinetics [23,24,25]. In fact, higher SFN excretion in urines after 24 h since consumption in null individuals rather than those with functional *GSTM1* was identified [25]. This evidence suggested that *GSTM1* positive individuals may have a different metabolism of SFN, with reduced SFN metabolites excretion. This may explain why *GSTM1* null individuals show less protection offered by SFN than positive subjects do [25].

In this work we studied the effect of SFN on interferon inflammation induced by cGAMP treatment, using a healthy immortalized human hepatocytes (IHH) cell line, focusing on *STING* and interferon signature modulation. The peculiar ability of SFN to induce the expression of phase II enzyme *GSTM1*, that plays an important role in its pharmacokinetics, was also evaluated in IHH cells. Moreover, as a secondary objective, we assessed the expression of *STING* and interferon signature in vivo in two healthy volunteers after the consumption of increasing doses of two commercial SFN supplements.

## 2. Results

### 2.1. Cytotoxicity of SFN on IHH Cells

**Figure 1:**
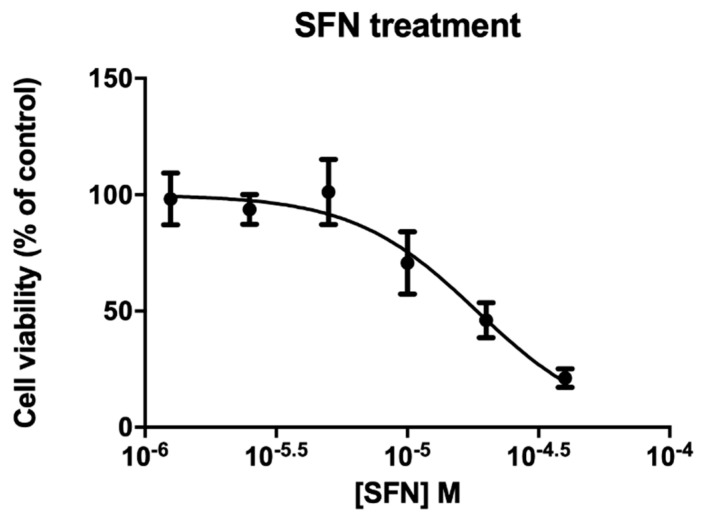
Cytotoxicity effects of sulforaphane (SFN) on IHH cell line. Cells were exposed for 72 h to SFN and cytotoxicity effects were analyzed by MTT assay. Data are reported as means ± SE of 3 independent experiments performed in triplicate. O.D.% observed to untreated cells.

To evaluate cytotoxicity of SFN on IHH cells, various concentrations were tested (1.25 × 10^−6^ M to 4 × 10^−5^ M) for 72 h by MTT assay. IHH cell line was found sensitive to SFN (EC_50_ 1.84 × 10^−5^ M, confidence intervals C.I. 1.37 × 10^−5^ M to 2.49 × 10^−5^ M) (Figure 1). The 10 µM concentration used for the subsequent treatment resulted in about 70% of cell viability.

### 2.2. STING Expression

**Figure 2:**
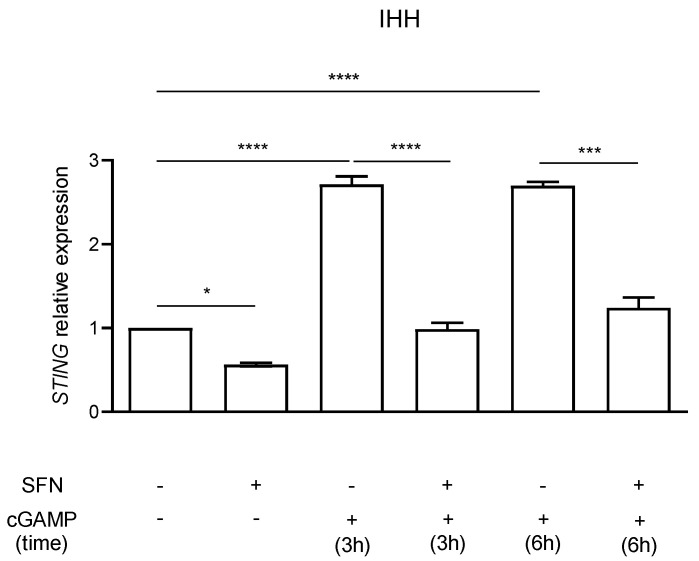
*STING* expression in IHH cells pretreated or not with sulforaphane (SFN, 10 µM) in presence of inflammatory stimulus cGAMP (5.9 µM). Data are shown as means and C.I. of two representative experiments and reported evaluating 2^−ΔΔCt^ values using untreated cells as calibrator and *HPRT1* and *G6DP* housekeeping genes as reference. *: *p* <0.05, one way ANOVA untreated CTRL IHH cells vs. 72 h SFN 10 µM treatment. ****: *p* < 0.0001, one way ANOVA IHH exposed to 5.9 µM cGAMP for 3 h and vs. IHH pre-treated with SFN 10 µM for 72 h and 5.9 µM cGAMP for 3 h. ***: *p* < 0.001, one way ANOVA IHH exposed to 5.9 µM cGAMP for 6 h vs. IHH pre-treated with SFN 10 µM for 72 h and 5.9 µM cGAMP for 6 h. ****: *p* < 0.0001, one way ANOVA untreated CTRL IHH cells vs. IHH treated with 5.9 µM cGAMP for 3 and 6 h.

*STING* expression in IHH cells was evaluated by RT-PCR. Cells were treated with 10 µM SFN in the presence or absence of the inflammatory stimulus 5.9 µM cGAMP added in the last 3 or 6 h of SFN incubation (Figure 2).

As expected, cGAMP induced a strong increase (*p* < 0.0001, one way ANOVA) in *STING* expression after 3 and 6 h of stimulation. A significant (*p* < 0.05, one way ANOVA) *STING* expression decrease was identified in cells treated with SFN for 72 h in comparison to the untreated control (CTRL) (Figure 2). The effect was even more evident when *STING* expression was induced after 3 h and 6 h of cell exposure to the inflammatory stimulus cGAMP in comparison to control. Cells pre-treated with SFN and stimulated with cGAMP showed a significant lower *STING* expression in comparison to those that were only stimulated with cGAMP (cGAMP 3 h vs. SFN+cGAMP 3 h *p* value < 0.0001; cGAMP 6 h vs. SFN+cGAMP 6 h *p* < 0.001, one way ANOVA) resulting in *STING* levels similar to the untreated control.

### 2.3. Interferon Signature Analysis of IHH Cells

**Figure 3:**
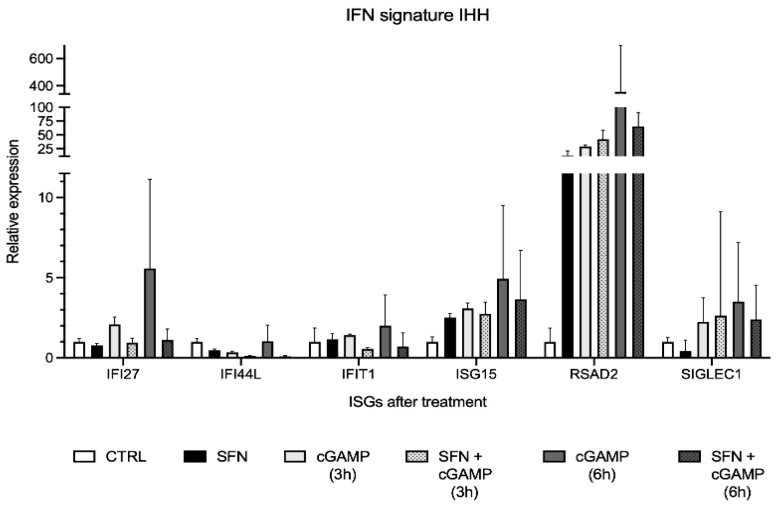
Interferon signature analysis of IHH cells pretreated with sulforaphane (SFN, 10 µM) for 72 h in presence of inflammatory stimulus cGAMP (5.9 µM) displayed as the expression levels of the six interferon-stimulated genes (ISGs). Data are shown as means and C.I. of one representative experiment and reported evaluating 2^−ΔΔCt^ values using untreated cells as calibrator (CTRL) and *HPRT1* and *G6DP* housekeeping genes as reference.

Interferon signature was analyzed on IHH cells treated with SFN 10 µM in presence and absence of the inflammatory stimulus cGAMP, to evaluate whether the expression of the six ISGs decreases in the presence of SFN during an inflammatory event (Figure 3).

An increase of ISGs expression after cell exposure to the cGAMP inflammatory stimulus was identified (Figure 3). In particular, the increment was higher after 6 h exposure in comparison to 3 h. Most ISGs showed a lower expression level in cells pretreated with SFN and subsequently exposed to cGAMP. In particular, the effect was more evident when cells were pretreated with SFN for 72 h and stimulated with cGAMP for 6 h in comparison to the same conditions at 3 h. Above all ISGs, *RSAD2* proved the most represented and overexpressed gene during cell stimulation with cGAMP and pretreatment with SFN for 72 h significantly reduced its overexpression. However, we noticed a strong increment in *RSAD2* expression also after SFN exposure alone, in absence of the cGAMP stimulus. Also, *ISG15* expression was slightly augmented by SFN exposure. The intensity of the interferon signature analyses is reported in Table 1 as IFN scores.

IFN score (Table 1) was higher in cells stimulated with the proinflammatory stimulus cGAMP. In particular, IFN score resulted about two times higher when the stimulus was maintained for a longer time. By contrast, the IFN score was similar, considerably lower in cells pre-treated with SFN and then stimulated with cGAMP for 3 or 6 h. Cells treated only with SFN have an IFN score similar to the control.

### 2.4. IHH GSTM1 Analysis

**Figure 4:**
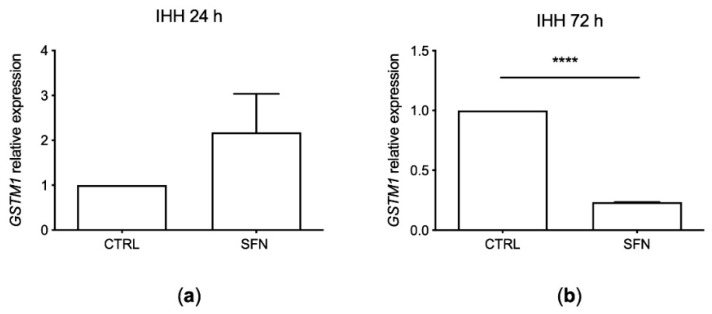
*GSTM1* expression in IHH cell line treated with sulforaphane (SFN, 10 µM) for 24 h (**a**) and 72 h (**b**). Data are shown as means and C.I. of three representative experiments and reported evaluating 2^−ΔΔCt^ values, using untreated cells as calibrator (CTRL) and beta-actin housekeeping gene as reference. ****: *p* < 0.001, one way ANOVA IHH exposed to SFN vs. untreated CTRL IHH cells.

GSTM1 expression was evaluated by RT-PCR after SFN exposure for 24 and 72 h at a concentration of 10 µM (Figure 4).

*GSTM1* expression increased after 24 h of treatment while significantly decreased after 72 h of SFN incubation (*p* < 0.001, one way ANOVA).

### 2.5. Basal STING Expression in Healthy Volunteers and Patients

**Figure 5:**
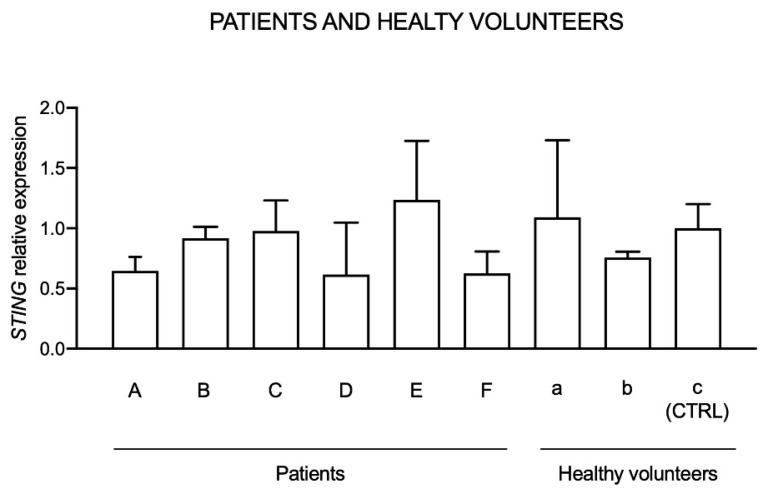
*STING* expression in patients (A–F) suffering from type I interferon dysregulations and healthy individuals (a–c). Data are reported evaluating 2^−ΔΔCt^ values, using healthy volunteer “c” as calibrator (CTRL) and *HPRT1* and *G6DP* housekeeping genes as reference.

Initially, basal *STING* expression in six patients suffering from type I interferon-related disorders (SLE, AGS, CANDLE like) and in three healthy individuals, not taking drugs or supplements, was evaluated to identify possible differences between healthy individuals and patients. The expression reported is normalized to a control (CTRL), which is represented by one of the three healthy individuals (Figure 5).

Results indicated that STING expression in patients and healthy volunteers is heterogenous and no relevant differences between healthy individuals and patients can be observed.

### 2.6. STING Expression after Administration of Lower-Dose SFN Supplement in HV1

**Figure 6:**
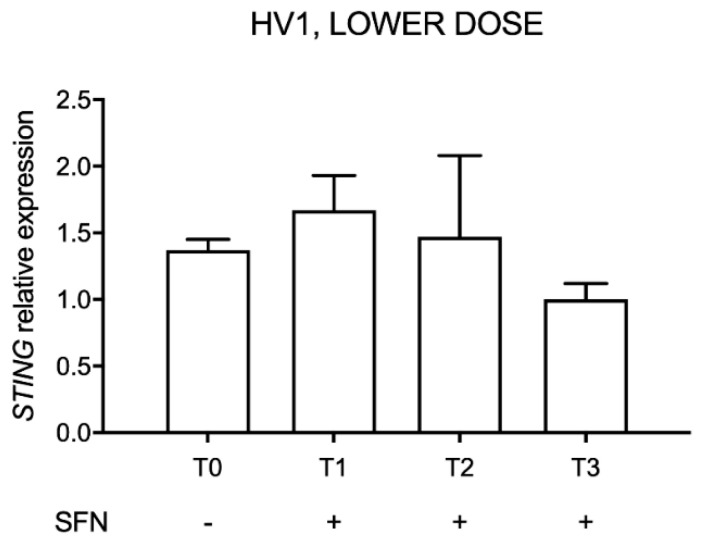
*STING* expression after sulforaphane (SFN) supplement consumption in the first volunteer (HV1). Data are reported evaluating 2^−ΔΔCt^ values considering the last day of assumption as calibrator (T3), using *HPRT1* and *G6DP* housekeeping genes as reference.

HV1 was treated with up to 25.2 mg of SFN daily for three consecutive days (T1: 24 h of treatment, T2: 72 h of treatment) and, after two weeks of interruption, for other seven days (T3) to evaluate whether oral SFN administration can decrease *STING* expression. The relative expression was evaluated considering *STING* expression measured in the last day of supplement intake as reference (T3) (Figure 6).

Results showed no significant changes in *STING* expression.

### 2.7. Interferon Signature Analysis after Administration of Lower-Dose SFN Supplement in HV1

**Figure 7:**
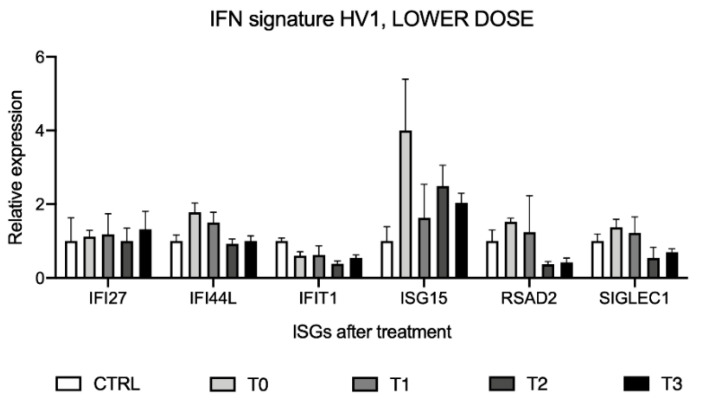
Interferon signature analysis after sulforaphane (SFN) supplement assumption in the first volunteer (HV1) displayed as the expression levels of the six interferon-stimulated genes (ISGs). Data are shown as means and C.I. of one representative experiment reported evaluating 2^−ΔΔCt^ values considering a pool of healthy volunteers’ cDNAs as calibrator and using *HPRT1* and *G6DP* housekeeping genes as reference.

To evaluate whether interferon signature analysis could be affected by oral SFN supplement administration, the expression levels of the six ISGs were assessed on the same samples used for *STING* analysis of the same volunteer (HV1), considering as calibrator a pool of healthy volunteers’ cDNAs (CTRL) (Figure 7).

Relative gene expression showed a reduction of the expression levels of most of the ISGs after SFN assumption, even if not significant. The intensities of interferon signature analyses, reported as IFN scores (Table 2), did not show significant changes for different intake times.

### 2.8. STING Expression after Higher-Dose SFN Supplement Administration in the Two Volunteers

**Figure 8:**
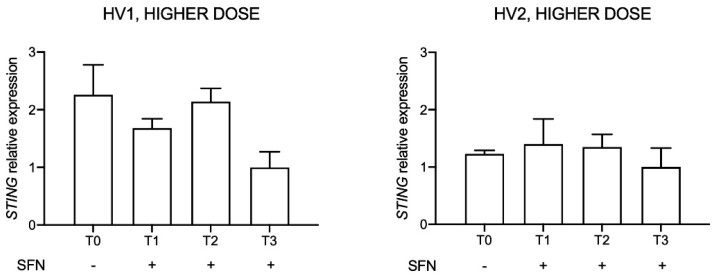
*STING* expression in two healthy volunteers (HV1, HV2) after sulforaphane (SFN) supplement administration at 90 mg/die for three days. Data are reported evaluating 2^−ΔΔCt^ values considering the last day of supplement consumption (T3) as calibrator and using *HPRT1* and *G6DP* housekeeping genes as reference.

After gene expression evaluation in HV1 taking lower supplement doses, we enrolled a second volunteer (HV2), increased the SFN dose and changed the commercial supplement (Broccoraphan^®^, Dieters). The dose was about four folds higher (90 mg/day) in comparison to the first one (up to 25.2 mg/day), and administration time lasted for three days (T1, T2, T3). Relative STING expression was evaluated before (T0) and after SFN assumption, considering as control the last day of supplement consumption (T3) (Figure 8).

A downward trend in *STING* expression was observed in HV1 but not in HV2.

### 2.9. Interferon Signature Analysis after Higher-Dose SFN Supplement Assumption in the Two Volunteers

**Figure 9:**
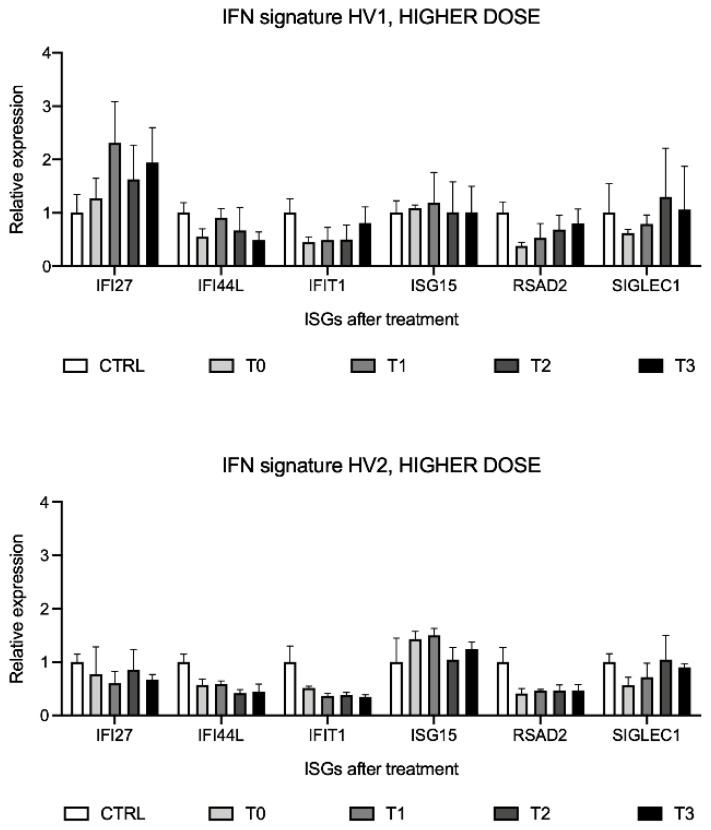
Interferon signature analysis in two healthy volunteers (HV1, HV2) after sulforaphane (SFN) supplement consumption at 90 mg/day for three days, displayed as the expression levels of the six interferon-stimulated genes (ISGs). Data are shown as means and C.I. of one representative experiment and reported evaluating 2^−ΔΔCt^ values considering a pool of healthy volunteers’ cDNAs as calibrator and using *HPRT1* and *G6DP* housekeeping genes as reference.

To evaluate the ISGs expression levels after consumption of a higher dose of SFN supplement (90 mg/day), interferon signature analysis was performed before (T0) and after SFN assumption (T1, T2, T3). ISGs expression results are relative to the calibrator, which is represented by a pool of healthy volunteers’ cDNAs (CTRL). Results did not highlight significant changes in ISGs after the 90 mg/day higher dose of SFN supplement in both volunteers (Figure 9).

The IFN scores did not show significant differences in both volunteers (Table 3 and Table 4).

### 2.10. Healthy Volunteers’ GSTM1 Genotype

To evaluate *GSTM1* genotype of the two healthy volunteers enrolled a genotype analysis was performed. Results indicated that the first volunteer (HV1) has a functional *GSTM1* gene while the second one (HV2) presents the deletion of the gene (null genotype).

### 2.11. GSTM1 Expression (HV1)

**Figure 10:**
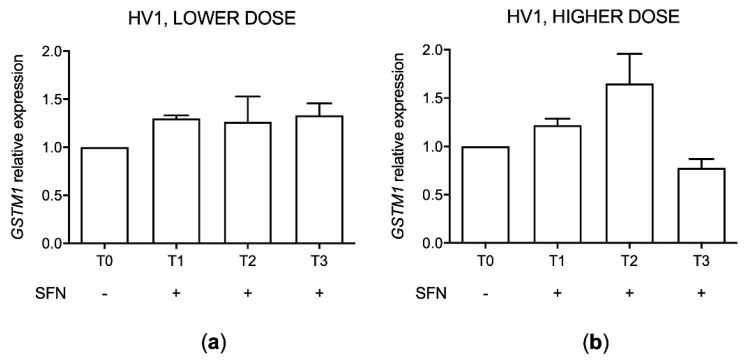
*GSTM1* expression in the first healthy volunteer (HV1) after sulforaphane (SFN) supplement consumption at 12.6 mg for the first day (T1), 25.2 mg for the second and third days (T2) and 25.2 mg for further seven days (T3) (**a**) and 90 mg/day for three days (**b**). Data are reported as 2^−ΔΔCt^ values considering the no assumption samples (T0) as calibrators and using *GAPDH* housekeeping gene as reference.

*GSTM1* expression was evaluated by RT-PCR in HV1 with the functional *GSTM1* genotype before and after SFN assumption. Analyses were performed before (T0) and after SFN assumption (T1, T2, T3) comparing the *GSTM1* level to no assumption condition (T0) both in lower and higher dose treatments (Figure 10).

An increment trend in *GSTM1* expression after SFN assumption in both treatment conditions was identified except for the last day (T3) of 90 mg dosage.

## 3. Discussion

Autoinflammatory diseases are rare genetic conditions defined by systemic inflammatory episodes caused by innate immune system activation in the absence of pathogens, with early onset in childhood. For these conditions, treatment is focused on main disease manifestations and includes various classes of drugs such as glucocorticoids, immunomodulatory agents, antimalarials and biological drugs [2,15,26]. These drugs are barely effective on type I interferon generating pathway, which is constitutively active in monogenic interferonopathies like AGS, and in multifactorial interferon-related disorders like SLE, dermatomyositis, Sjögren syndrome and others [6]. In this regard, SFN, a small molecule derived from vegetables, may be useful as a possible support to dampen interferon inflammation given its ability to modulate the expression of *STING* [20], an upstream pathway component that mediates type I interferon production. Based on this evidence, we evaluated SFN effect in vitro in the hepatic IHH stable cell line and in vivo on two healthy volunteers.

After SFN exposure for 72 h, a decrease in *STING* expression in comparison to the untreated control was detected in IHH cells. This result confirmed the ability of SFN to reduce its effects in terms of *STING* expression in vitro, as described by Olagnier and colleagues work in the THP-1 human monocytic cell line after SFN treatment [20]. When the IHH cells were treated with the interferon inducer cGAMP, the treatment with SFN was able to prevent the stimulation-induced increase in *STING* expression. Similarly, we examined the so-called interferon signature, a set of characteristic overexpressed interferon-stimulated genes in patients suffering from type I interferonopathy. Interferon signature showed a similar behavior, demonstrating a considerably higher IFN score when cells were exposed to the cGAMP inflammatory stimulus with more than halved IFN score in cells pretreated with SFN and in presence of cGAMP. Therefore, our study provides additional information about *STING* expression in the presence of an inflammatory stimulus with and without pretreatment with SFN. These data provide knowledge about the role of SFN in interferon-driven inflammation suggesting a SFN preventing effect on inflammatory stimuli on IHH cell line.

Moreover, we analyzed the SFN effect on *GSTM1*, a gene encoding for a phase II xenobiotic detoxification enzyme, in IHH cells, previously confirmed wild type. In IHH cells we found an increased *GSTM1* expression after 24 h, while a decrease after 72 h using a 10 µM concentration of SFN that resulted in a 30% of cell cytotoxicity. The modulation of SNF on gene expression depends on its concentration which can cause both antioxidant and pro-oxidant effects with consequent modulation on *GSTM1* expression [27]. This result seems in line with the literature [27] where slightly toxic SFN concentrations can increase *GSTM1* levels due to the generation of ROS that interact with NRF2 pathway which can modulate key components of inflammation-promoting gene expression contrasting the action of nuclear factor NF-κB. Instead, after 48 h, when ROS was significantly reduced and could not have an effect on NRF2, *GSTM1* expression also decreased.

Interestingly, in the work of Yoon-Jin Lee and colleague [27] demonstrated that SFN increases the nuclear translocation of NRF2 via a ROS dependent mechanism in bronchial epithelial cells at a concentration of 10 µM corresponding to around 30% of cytotoxicity: the paper describes a rapid increase in intracellular ROS levels which rapidly starts within 10 min after SFN addition, peaks at 8 h and gradually declines until 48 h [27]. From this result, it is possible to assume that as the ROS peak was found 8 h after treatment, and gradually decreased until 48 h with a consequent decrement of NRF2 nuclear accumulation, also *GSTM1* induction probably could cease explaining our result of lower *GSTM1* expression after 72 h of treatment using a slight cytotoxic dose of SFN.

As a secondary objective, we performed an exploratory analysis on *STING* expression levels and IFN score after SFN assumption in two healthy volunteers, to assess the possibility of extending the study by enrolling an adequate number of subjects. After preliminary data obtained in one volunteer with low SFN doses (up to 25.2 mg/day), showing no significant effect on *STING* expression and IFN score, we increased the supplement dose (90 mg/day for three days), choosing a commercial supplement whose SFN content and indications resulted convenient to our purpose (Broccoraphan^®^, Dieters). At the same time, we enrolled a second volunteer (HV2) in order to evaluate potential individual differences in response. At the same time, we performed a genotype analysis of *GSTM1* since *GSTM1* genotype has an important role in SFN pharmacokinetics: in particular, *GSTM1* null or not functional individuals have a greater excretion of SFN and its metabolites in the first 24 h after SFN assumption in comparison to individuals with a functional gene [25]. Results were not consistent between the two subjects. In particular, only HV1 showed some *STING* expression decrease after treatment. In our experiment, HV1 was found to be wild type for *GSTM1*, while HV2 turned out not functional. This fact may support an interpretation of the different STING expression response to SFN in the two volunteers: the *GSTM1* functional one had a more notable decrease trend in comparison to the *GSTM1* non-functional individual. This result may suggest that, if SFN excretion is not rapid, as in *GSTM1* functional individuals, *STING* repression can be possible, in a dose and time dependent manner, according to Olagnier and colleagues [20]. In fact, the lower *STING* level was appreciable at the last day of assumption at higher doses (90 mg/day) only in the HV1 with the functional *GSTM1* gene. Regarding the SFN effect on *GSTM1*, we can appreciate an upregulation after both treatment conditions in the HV1 except for the last day of the 90 mg dose assumption. The induction of *GSTM1* by SFN is in line with the above reported results on the IHH line where, as the initial induction of *GSTM1* gradually ceases for the depletion of ROS and nuclear accumulation of NRF2, leading to the reduction of the *GSTM1* expression in the last day of SFN assumption.

The treatment with SFN did not impact the IFN score in the two volunteers. However, considering that healthy individuals did not present an inflammatory state and ISGs are downstream from *STING* and dependent on type I interferon production, results are reasonable, although this is a preliminary experiment. Thus, the real anti-interferon potential of SFN could be difficult to assess in unstimulated conditions.

The presence of other antioxidants could be a confounder in our analysis. However, beyond SFN, only the first supplement used (Broccoli Sprout, Love Life) also contained other antioxidants such as polyphenols. However, the much higher bioavailability of SFN in comparison to poorly bioavailable polyphenols [17], together with the data acquired using the second supplement devoid of other antioxidants, suggests that the effects highlighted in healthy donors are SFN-driven.

## 4. Materials and Methods

### 4.1. Cell Culture

The immortalized human hepatic IHH cell line was maintained in Dulbecco’s modified Eagle’s medium (DMEM) high glucose with the addition of 10% fetal bovine serum (Sigma-Aldrich, St. Louis, MO, USA), 1.25% L glutamine 0.2 M (EuroClone, Milan, Italy), 1% penicillin 0.03 M (EuroClone), streptomycin 0.02 M (EuroClone), 1% Hepes buffer 1 M (EuroClone), 0.01% human insulin 10^−4^ M (Sigma-Aldrich), and 0.04% dexamethasone 2.1 × 10^−3^ M (Sigma-Aldrich). Cell cultures were maintained according to standard procedures in a humidified incubator at 37 °C and with 5% CO_2_, and cell passage was performed twice a week.

### 4.2. IHH Treatment

IHH cells (5 × 10^3^ cells/well) were exposed for 72 h to different concentrations of sulforaphane (SFN) (1.25 × 10^−6^ M to 4 × 10^−5^ M) for cytotoxicity analysis evaluated by the MTT assay.

IHH cells were treated with SFN 10 µM for 24 h and 72 h in presence and absence of the pro-inflammatory stimulus cGAMP (Table 5) for *STING* and for interferon signature analysis. In particular, cGAMP 5.9 µM was added in the last 3 or 6 h of incubation in presence or absence of SFN 10 µM.

### 4.3. Cytotoxicity Assay

In the last 4 h of treatment, a solution of 3-(4,5-dimethylthiazol-2-yl)-2,5-diphenyltetrazolium (MTT) was added (final concentration 0.5 mg/mL) and the crystals produced by metabolically active cells were solubilized with 100 μL of DMSO. The absorbance was read by an Automated Microplate Reader EL 311s (Bio-Tek Instruments, Winooski, VT, USA) at 540/630 nm. Data are the means ± SE of at least three independent experiments performed in triplicate and are reported as % of untreated controls (absorbance treated/absorbance untreated control × 100).

### 4.4. Healthy Volunteers

Two individuals without clinical symptoms of diseases were selected for the study: both were of Caucasian ethnicity, one male and one female, more than 18 years old.

At first, one healthy volunteer (HV1) assumed up to 25.2 mg/day SFN (Broccoli Sprout Extract^®^, Love life, Cambridge, UK) for three consecutive days (T1: 24 h of assumption, T2: 72 h of assumption) and, after two weeks of stop, for other seven days (T3). Doses were decided considering the steady state concentration of SFN, which, taking 8.2 mg every 8 h resulted in the 0.3–0.6 µM range [28,29]. In particular, 4.2 mg of SFN supplement was administered every 8 h for the first 24 h (12.6 mg daily), 8.2 mg every 8 h for the next 48 h (25.2 mg daily) and, after two weeks of stop, the same dosage for 7 days. Broccoli sprout extract^®^ Love life supplement was chosen because the amount (2.5 mg of SFN/capsule 600 mg) of SFN contained in each capsule was convenient to reach pharmacologically relevant blood concentrations of the drug. Peripheral blood samples were collected for RNA extraction before treatment (T0 no assumption), at T1, T2, and at T3 (Table 6).

In the second treatment, both volunteers (HV1, HV2) assumed an almost four-times-higher dose (90 mg daily for 3 days). This time, the supplement chosen was *Broccoraphan^®^* (Dieters, Frechen, Germany) because the formulation was convenient in relation to our objectives, despite the bad taste of the supplement powder (~15 mg SFN/g supplement—90 mg corresponds to 6 g daily). Indeed, the dose should lead to an SFN steady state concentration of 1–2 µM [28,29] and was considered safe, as reported by the supplement indications. Peripheral blood samples were collected for RNA extraction before treatment (T0 no assumption), at T1, T2, and T3 for both volunteers (Table 7).

### 4.5. RNA Extraction

Total RNA of the IHH cell line was extracted with TRIzol reagent (Invitrogen, Waltham, MA, USA) while healthy volunteers’ total RNA was extracted using the PAXgene Blood RNA kit (PreAnalytiX, Hombrechtikon, Switzerland) according to the manufacturer’s instruction. The obtained RNA was quantified using Nanodrop 2000 spectrophotometer (ThermoFisher Scientific, Waltham, MA, USA) and reversed-transcribed into cDNA using the High-Capacity RNA-to-cDNA kit (Applied Biosystem, Waltham, MA, USA).

### 4.6. Real-Time PCR

#### 4.6.1. Interferon Signature and STING Analysis

Interferon signature analysis was performed by relative quantification of six interferon-stimulated genes (ISGs: *IFI27*, *IFI44L*, *IFIT1*, *ISG15*, *RSAD2*, *SIGLEC1*) by real-time PCR (RT-PCR), using UPL Probes (Roche, Basel, Switzerland)*,* TaqMan Gene Expression Master Mix (Applied Biosystems), and a AB 7500 Real Time PCR system (Table 8). The same analysis was also used for *STING* and the housekeeping reference genes *HPRT1* and *G6PD*.

The RT-PCR protocol consists of an initial denaturation for 2 min at 50 °C and for 95 °C for 10 min, followed by 40 cycles of heating at 95 °C (15 s), and then a final extension for 1 min at 60 °C. Data were analyzed with 7500 SDS (Applied Biosystems) analysis software. Data were normalized with two housekeeping genes: *G6DP* and *HPRT1*. Relative quantification was performed using the 2^−ΔΔCt^ method using as reference the cDNA mix of 10 healthy controls (CTRL) for ISGs while cDNA of the last day of treatment sample (T3) for *STING.* For interferon signature analysis, the IFN score was calculated through the median of the relative quantifications of the six ISGs.

#### 4.6.2. GSTM1 Expression

RT-PCR was performed using the TaqMan^®^ Gene Expression Assays (Hs01683722_gH, Applied Biosystems) in a Thermal Cycler Dice Real Time System (BIO-RAD). Relative quantification is represented as 2^−ΔΔCt^ with respect to the housekeeping genes beta-actin (*ACTB*) and glyceraldehyde 3-phosphate dehydrogenase (*GAPDH*), setting untreated IHH and untreated donor as reference. The RT-PCR protocol for *GSTM1* analysis consists of an initial denaturation for 10 min at 95 °C, followed by 40 cycles of heating at 95 °C (15 s) and 60 °C (1 min). All experiments were carried out in duplicate and the reproducibility of the observations was confirmed in two or three independent experiments.

#### 4.6.3. GSTM1 Genotype

*GSTM1* genotype state was assessed by TaqMan^®^ CNV genotyping Assays (Applied Biosystems) kit, containing forward and reverse specific primers for the interested amplicon, which, in that case, is *GSTM1* and a FAM conjugated probe. Additionally, TaqMan™ Copy Number Reference Assay, human RNase P was used to assess the presence of a double copy gene. In this case, too, the kit contains forward and reverse specific primers for the interested amplicon (human RNase P) and a VIC^®^ dye–labeled TAMRA^™^ probe. The thermic protocol used was 95 °C for 10 min, 95 °C for 15 sec, and 60 °C for 1 min. The whole reaction was repeated for 40 cycles. Results are expressed as deleted vs. functional *GSTM1*.

### 4.7. Statistical Analysis

Results are presented as mean ± 95% confidence intervals (C.I.) from up to two independent experiments. Statistical analyses were performed using GraphPad Prism software (version 8.0.2). One-way ANOVA and Bonferroni’s post-test were used for gene expression analysis. *p* values < 0.05 were considered statistically significant.

## 5. Conclusions

In conclusion, this study confirmed SFN inhibiting action on inflammatory stimuli response in vitro in terms of *STING* reduction and ISGs expression. We observed a *STING* reduction trend in vivo only in the HV1 after SFN administration. The higher efficacy of SFN in *STING* reduction could be related to the functional *GSTM1* genotype related to a slower elimination rate and therefore a higher SFN efficacy. Since individuals were healthy and not affected by inflammatory conditions, no effect of SFN treatment was observed on ISGs expression, as already characterized by low ISGs expression. From these premises, it will be reasonable to expand the study to a larger group of healthy individuals to further investigate the association between *STING* modulation by SFN and *GSTM1* genotype, and subsequently to evaluate these effects in patients with type I interferonopathies.

## Figures and Tables

**Table 1:** IFN scores of IHH cells treated with SFN and cGAMP.

CTRL	SFN 72 h	cGAMP 3 h	SFN 72 h + cGAMP 3 h	cGAMP 6 h	SFN 72 h + cGAMP 6 h
1.00	0.96	2.16	1.79	4.22	1.75

CTRL = untreated control; SFN = sulforaphane 10 µM; cGAMP = 5.9 µM.

**Table 2:** IFN scores calculated through the median of the relative quantifications of the six genes of the interferon signature analysis after administration of lower-dose sulforaphane (SFN) supplement in the first healthy volunteer (HV1).

CTRL	T0	T1	T2	T3
1.00	1.44	1.23	0.73	0.85

**Table 3:** IFN scores calculated through the median of the relative quantifications of the six interferon-stimulated genes (ISGs) of the interferon signature analysis after administration of higher-dose sulforaphane (SFN) supplement in the first healthy volunteer (HV1).

CTRL	T0	T1	T2	T3
1.00	0.58	0.85	0.84	0.91

**Table 4:** IFN scores calculated through the median of the relative quantifications of the six interferon-stimulated genes (ISGs) of the interferon signature analysis after administration of higher-dose sulforaphane (SFN) supplement in the second healthy volunteer (HV2).

CTRL	T0	T1	T2	T3
1.00	0.57	0.60	0.66	0.57

**Table 5:** Different combination treatment of SFN and cGAMP used on the IHH cell line.

SFN	cGAMP	Exposure Time
10 µM	-	24 h
10 µM	-	72 h
-	5.9 µM	3 h cGAMP
-	5.9 µM	6 h cGAMP
10 µM	5.9 µM	SFN 72 h, 3 h cGAMP
10 µM	5.9 µM	SFN 72 h, 6 h cGAMP

SFN = sulforaphane; cGAMP = cyclic guanosine monophosphate–adenosine monophosphate.

**Table 6:** Doses and timing of sulforaphane (SFN) administrations in the first healthy volunteer enrolled (HV1).

Lower Dose	
Dose/Day	Administration Timing
-	No assumption (T0)
12.6 mg	Day 1 (T1)
25.2 mg	Day 2–3 (T2)
25.2 mg	7 days assumption (T3)

**Table 7:** Doses and timing of sulforaphane (SFN) administrations in both volunteers enrolled (HV1, HV2). The second-round dose treatments were considered high doses and referred as “higher dose”.

Higher Dose	
Dose/Day	Administration Timing
-	No assumption (T0)
90 mg	Day 1 (T1)
90 mg	Day 2 (T2)
90 mg	Day 3 (T3)

**Table 8:** Universal Probe Library (UPL) probes (Roche) and primers (Eurofins Genomics) for the six interferon-stimulated genes (ISGs) of interferon signature and STING analysis by Real-Time PCR.

Probes and Primers			
GENE	Probe (10 μM)	Primer Forward (20 μM)	Primer Reverse (20 μM)
**ISGs assessed for interferon signature analysis**			
*IFI27*	P. n. 21	GTGGCCAAAGTGGTCAGG	CCAATCACAACTGTAGCAATCC
*IFI44L*	P. n. 15	TGACACTATGGGGCTAGATGG	TTGGTTTACGGGAATTAAACTGAT
*IFIT1*	P. n. 82	TCCACAAGACAGAGAATAGCCAGAT	GCTCCAGACTATCCTTGACCTG
*ISG15*	P. n. 76	GAGGCAGCGAACTCATCTTT	AGCATCTTCACCGTCAGGTC
*RSAD2*	P. n. 76	ACAAATGCGGCTTCTGTTTC	GAAATGGCTCTCCACCTGAA
*SIGLEC1*	P. n. 76	CTGCCCTGCAAGTCCTCTA	CAGCAGGTGGCTCACTGTC
**Primers and probe for measuring *STING* expression**			
*STING*	P. n. 51	CGCCTCATTCCCTACCAG	TGCCCACAGTAACCTCCTCC
**Housekeeping genes**			
*HPRT1*	P. n. 73	TGACCTTGATTTATTTTGCATACC	CGAGCAAGACGTTCAGTCCT
*G6PD*	P. n. 82	GCAAACAGAGTGAGCCCTTC	GAGTTGCGGGCAAAGAAGT

## Data Availability

Raw data will be provided upon reasonable request.
